# ALTE and Feeding Intolerance as a Presentation of Double Aortic Arch

**DOI:** 10.1155/2016/8475917

**Published:** 2016-09-18

**Authors:** Rekha Krishnasarma, Liza Green Golan Mackintosh, Francine Bynum

**Affiliations:** Department of Pediatrics at the Children's Hospital of Los Angeles, Los Angeles, CA, USA

## Abstract

Many children who are admitted to pediatric hospitals with the chief complaint of apparent life-threatening event (ALTE) are, in fact, well appearing by the time the inpatient medical team evaluates the patient. This presents a diagnostic and therapeutic challenge. We describe a case of a six-month-old full-term female presenting with an ALTE and found to have a double aortic arch, a congenital anomaly that usually presents with a more progressive onset of symptoms such as chronic cough, positional stridor, and feeding difficulties. This case highlights the importance of maintaining a broad differential in a patient presenting with findings of tracheoesophageal pathology on clinical exam.

## 1. Introduction

We discuss the case of a 6-month-old full-term female with long-standing feeding intolerance and chronic respiratory symptoms presenting with an apparent life-threatening event and found to have a double aortic arch.

## 2. Case Presentation

A 6-month-old full-term female with a history of respiratory syncytial virus bronchiolitis at two weeks of age presented to our hospital via emergency medical services with the chief complaint of ALTE. As per the mother, the patient was noted to have an episode of perioral cyanosis, coughing, and increased work of breathing after being placed on her back immediately following a bottle-feed. When the mother noted these symptoms, she picked up the patient who was described as “pale” and “limp.” A bystander was able to forcefully pat the patient's back and formula was expressed from the mouth. By the time medical personnel arrived, the patient was back to her baseline behavior.

Upon further questioning, it became clear that the patient had a several month history of chronic cough, congestion, and feeding intolerance for which she had already undergone an extensive outpatient workup. This workup included testing for various immunodeficiencies, as well as a laryngoscopy by otolaryngology. Per report, these tests and the above procedure did not reveal any pathology. The mother reported that the primary care physician had seen the patient earlier in the week of presentation for increased coughing and prescribed nebulized albuterol treatments and a short course of steroids for presumed reactive airway disease. Neither intervention alleviated her symptoms. While this was the patient's first episode of cyanosis associated with cough, the mother reported a worsening of the patient's cough and shortness of breath upon lying flat and after feeding.

After admission to the floor, the team observed the patient feed which resulted in a choking-like episode and overt respiratory distress, only alleviated by positional maneuvers. Other pertinent positives on her physical exam were baseline audible breathing with occasional inspiratory stridor. Given the patient's increased symptomatology with feeding and her concerning baseline respiratory exam, she was deemed unsafe for oral feeds until her evaluation was complete.

**Figure 1:**
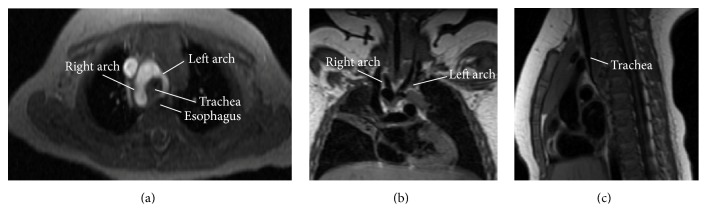
Contrast-enhanced magnetic resonance angiography demonstrating the double aortic arch with dominant right arch as demonstrated on axial (a), coronal (b), and sagittal planes (c). The axial and sagittal images reveal that the trachea and esophagus are surrounded and compressed by a vascular ring formed by the double aortic arch. The coronal image demonstrates that the right arch is larger and higher in position than left counterpart, which is hypoplastic.

Our initial evaluation included a chest radiograph and gastric emptying scintigraphy. The chest radiograph demonstrated slightly hyperinflated lung fields bilaterally but was otherwise unremarkable. The gastric emptying study revealed delayed gastric emptying, without overt evidence of reflux. Additional workup for potential anatomic abnormalities was pursued with fluoroscopy of the upper gastrointestinal tract, which showed a prominent posterior impression on the esophagus on the lateral projection at the level of the aortic knob. On the frontal view, the esophageal impression was more exaggerated on the left side compared to the right. These findings suggested an aberrant right subclavian artery, or double aortic arch, and the radiologist recommended magnetic resonance imaging (MRI) to better elucidate these findings (Figure 1). MRI of the chest revealed a double aortic arch with the right arch being dominant, significantly larger, and more superior in position than its left counterpart, which was hypoplastic. The left carotid and left subclavian arose from the smaller left arch. There was marked narrowing of the trachea at the site of the vascular ring; the tracheal diameter measured approximately 2.5 mm just above the carina. With the diagnosis of a double aortic arch compressing the trachea and esophagus, our patient was transferred to the cardiothoracic team for surgical repair with video-assisted thoracoscopic surgery (VATS). She underwent division of left arch, leaving the dominant right arch in place.

## 3. Discussion

An apparent life-threatening event (ALTE) is defined as an episode that is frightening to the observer and is characterized by some combination of apnea, color change, and choking or gagging [1]. The differential diagnosis for the etiology of ALTEs in infants includes a broad array of congenital or acquired disorders and spans multiple organ systems. ALTE is associated with infectious or inflammatory pulmonary conditions, gastroesophageal reflux, neurologic disease, immune compromise, metabolic disorders, and congenital anomalies [2]. While rare, congenital anomalies such as vascular rings must be considered in an infant who presents with an ALTE preceded by a history of chronic symptomatology of respiratory and/or feeding difficulties, as depicted by our case.

Vascular rings arise when specific portions of the paired aortic arch system persist or regress abnormally. They can be complete or incomplete depending on the compression of the trachea and esophagus, with complete vascular rings encircling and compressing the two structures. The double aortic arch, a complete vascular ring, is the most common type, resulting from the failure of the fourth embryonic branchial arch to regress, leading to an ascending aorta that divides into a left and right arch that fuse together to completely encircle the trachea and esophagus [3, 4].

Double aortic arches comprise 1-2% [5] of all cardiac anomalies and up to one-third of all vascular rings [6, 7]. Symptoms usually appear in the first six months of life. Compression from complete anomalies leads to respiratory and gastrointestinal symptoms. Airway symptoms such as inspiratory stridor and wheezing are found more commonly when the trachea is compressed in a double aortic arch, while gastrointestinal symptoms such as dysphagia, reflux, and choking episodes [6] are seen when the esophagus is compressed posteriorly. Respiratory arrest has been reported in 7% of reported cases [8]. Not surprisingly, complete anomalies are more often associated with more severe symptoms than incomplete forms, making a double aortic arch the most common vascular ring that necessitates surgical repair.

Cross-sectional imaging with magnetic resonance angiography (MRA) or computed tomography angiography (CTA) of the chest [9] is crucial in the definitive diagnosis and surgical management of vascular rings. Generally, a patient has had abnormalities found on chest X-ray, echocardiography, bronchoscopy, gastric emptying studies, or fluoroscopic studies such as a modified barium swallow or upper gastrointestinal series. In our case, the upper gastrointestinal series showed compression of the trachea and esophagus, thus prompting further evaluation with MRA.

Surgery to correct a double aortic arch is reportedly well tolerated [10]. Two approaches are described, via a left thoracotomy and/or VATS [11]. In repair of a double aortic arch, the division is made in the hypoplastic arch segment, which is usually the left. Complications and deaths during or after this repair have been reported in patients who have had other comorbid medical conditions. Operative mortality for vascular ring division is close to zero as demonstrated in the pediatric surgery literature [11].

In conclusion, our clinical case demonstrates the importance of recognizing symptoms and physical findings of vascular rings. Although this patient presented with an acute event, she demonstrated repeated progressive episodes of postprandial choking and chronic respiratory distress, prompting additional workup. With advances in both radiologic diagnosis and surgical management, the expedient diagnosis and treatment of the double aortic arch is possible. Needless to say, thorough history taking and physical exam skills are of utmost importance as well as recognition of the need for further diagnostic examinations.
